# Effect of Process Variables and Ingredients on Controlled Protein Network Creation in High-Moisture Plant-Based Meat Alternatives

**DOI:** 10.3390/foods12203830

**Published:** 2023-10-19

**Authors:** Animesh Singh Sengar, Michael Beyrer, Ciara McDonagh, Uma Tiwari, Shivani Pathania

**Affiliations:** 1 Food Industry Development Department, Teagasc Food Research Centre, Ashtown, D15 DY05 Dublin, Ireland; animesh.sengar@teagasc.ie (A.S.S.); ciara.mcdonagh@teagasc.ie (C.M.); 2 School of Food Science and Environmental Health, Technological University Dublin, City Campus, Central Quad, Grangegorman, D07 ADY7 Dublin, Ireland; 3 Institute of Life Technologies, University of Applied Sciences and Arts Western Switzerland, 1950 Sion, Switzerland; michael.beyrer@hevs.ch

**Keywords:** high-moisture extrusion, extrusion mechanism, alternative proteins

## Abstract

The market has observed a rapid increase in the demand for plant-based foods as an alternative to animal meat products. Technologies such as high-moisture extrusion (HME) have the potential to develop anisotropic structures using alternative protein ingredients. This article discusses the different possible mechanisms responsible for structure formation and the effect of extrusion process parameters and outlines the recent advances in the long cooling dies (LCDs) used for meat alternative development. The role of different protein ingredients and the impact of combining them with other biopolymers were also evaluated. The underlying mechanism behind anisotropic structure formation during HME is a synergistic effect, with substantial dependence on the source of ingredients and their processing background. Formulation including proteins derived from plants, insects, animals, and microalgae with other biopolymers could pave the way to develop structured meat alternatives and fill nutritional interstices. Dynamic or rotating annular gap cooling dies operating at freely controllable shear and static annular gap dies are recent developments and assist to produce layered or fibrous structures. The complex chemical sites created during the HME of plant protein favour flavour and colour retention. This paper summarises the recent information published in the scientific literature and patents, which could further help researchers to fill the present knowledge gaps.

## 1. Introduction

Meat alternatives, also known as “meat substitutes/analogues” but not confined to that, have gained substantial commercial popularity among consumers due to a diet shift towards veganism/vegetarianism [1]. Not only are these products processed to mimic a meat-like texture, mouthfeel, appearance, and flavour, but they are also designed to hold a similar nutritional profile to conventional meat products. It has been established that consumer acceptance of plant-based meat alternatives has increased due to their healthfulness, high-quality protein, and agreeable taste [2]. Driving factors influencing this dietary shift towards plant-based products are (a) limited resources of protein for a growing population, (b) energy and protein feed-to-food conversion inefficiency, (c) the antibiotics use in animal production, (d) greenhouse gas emissions, (e) land and water usage in animal production systems, and (f) the risk of pathogenic bacteria and infections from zoonotic viruses in animal meat [3,4,5,6]. Animal-based food (including livestock feed) contributes to 57% of the total greenhouse gas produced during the whole food production chain [5].

Plant-based meat alternatives have been consumed worldwide for a long time. Although there are many routes to developing meat alternatives, acquiring meat-like textures is incredibly challenging and empirical due to our limited understanding of protein structuring mechanisms and protein techno-functionality, along with underdeveloped efficient manufacturing process control. Meat alternative development entails the combination of native and modified ingredients to attain adequate portions of essential amino acids in the human diet with improved digestibility [7]. Plants, aquatic biomass, cellular agriculture, and insects are growing in popularity in the current marketplace as alternative protein sources [8,9]. Soybeans, wheat, oats, peas, faba beans, and rapeseed are among the most common plant-based sources used as animal protein alternatives [10,11,12,13,14,15]. Several functional ingredients/additives are added to the formulation to acquire a desirable texture and flavour profile. Structure formation and integrity, as well as the appearance of meat alternatives, have been improved using novel functional ingredients such as banana floret, jackfruit flour, oyster mushrooms, cassava, and coconut flour. These ingredients have been used in the formulations for vegan sausage, soy-based meat alternatives, as well as a filler in chicken-based sausage [16,17,18]. Jackfruit sandwiches could be a substitute for pulled pork sandwiches, but the much lower protein content in jackfruit contests its use as an apt ingredient for meat alternatives [19]. Therefore, adding such ingredients favours the structure formation and their role could be better understood in the present review. To develop a fibrous structure, it is important to understand ingredients’ functionality and behaviours under specified processing conditions (largely dependent on the method of production).

**Figure 1:**
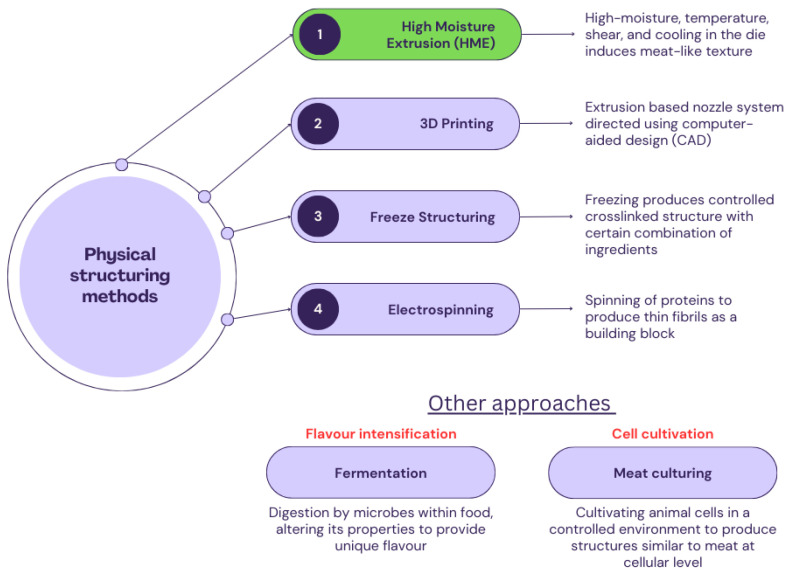
Overview of different meat alternative developing methods.

Different methods (Figure 1) such as high-moisture extrusion (HME), 3D printing, freeze structuring, and electrospinning are used for physical structuring. Whereas, fermentation and cultured meat techniques are the other explored approaches to developing a high-quality meat alternative product [20,21,22,23,24,25,26]. Plant-based ingredients have been traditionally fermented for centuries to serve affordable, nutritious, and appealing processed protein-rich products. Examples of commercially available fermented products are tofu, tempeh, kinema, yuba, risofu, and remis algen [27]. However, the distinct flavour associated with these products, allergic reactions to their critical ingredients, i.e., soy/wheat, and their associated environmental footprints limits their acceptance in the broader market. Further, the different methods to develop meat alternatives are relatively novel and widely under development. 3D printing/fused deposition modelling uses an extrusion-based nozzle system to develop a customized product with the desirable quality attributes [28]. The products developed thereof mimic a meat-like texture and mouthfeel; however, the printability of food ingredients is relatively limited and high investment costs are the constraints associated with this technology. Another approach adopted by Nieuwland et al. [24] was the use of electrospinning (spinning of proteins) technology for the production of thin fibrils as the building blocks of meat substitutes. The research group suggested that the electrospun material, when aligned in order, could potentially mimic a meat-like texture. A freeze structuring technique has also been used to produce spongy structures, which might suit for restricted product applications [23]. This technique involves a series of phenomena via ice crystal formation and their growth in the protein gel, phase separation, and the cold denaturation of proteins to produce densely packed layered structures. The freeze structuring of plant protein is much dependent on the composition of ingredients, which requires further investigation [29]. Another technique that has gained attention in the meat alternative landscape is cultured meat technology. It uses a bottom-up strategy which starts with assembling building blocks (sourced from animals) to mimic animal meat tissues; therefore, calling it a meat alternative could be criticised. Although the above-mentioned technologies have been used to develop meat alternatives successfully, there is, however, only one technology, i.e., high-moisture extrusion (HME), that is available for large-scale manufacturing and has revolutionised the meat alternative market globally.

**Figure 2:**
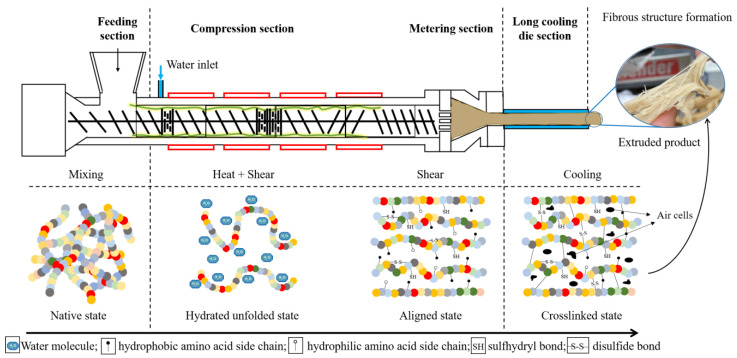
Structural and functional modifications of protein during HME-based fibrous structure formation.

The HME process operates at a feed moisture content > 40% and an elevated barrel temperature (~100 °C), followed by the immediate cooling of constituents to develop a cross-linked, elongated, fibrous (also known as anisotropic) network in plant-based ingredients [14,30]. HME induces a variety of reactions in multicomponent systems such as starch gelatinization, molecular degradation, and protein–protein and protein–polysaccharide interactions (Figure 2). The protein structuring is influenced by the realignment of structures in the mixing zone (α-helix and β-sheet), the unfolding of the protein molecular chain, and the formation of hydrogen bonds (disruption of β-turn) due to the high temperature, shear, and pressure in the extruder [31]. The extrusion parameters influence the reactions at the molecular level and affect the rheological properties. It has been established that the extrusion parameters, i.e., temperature–time profiles, screw speed, and die design, highly correlate with the extrudate quality. Twin-screw extrusion is classified as a top-down technology which produces a meat-like structure at a macroscale with a distinct capability of molecular level modification, as well as the micro-structuring of protein molecules [32].

There is also an increasing interest in using a combination of techniques to create nutritious and textured meat alternatives, such as fermenting textured vegetable protein using *Bacillus subtilis* [26]. Fermentation-enabled cultured meat is enhanced in taste, texture, post-production nutrition, product reproducibility, and shelf life [27]. Combined techniques could enhance the physical and structural characteristics, nutritional value, and digestibility of the end product.

Therefore, the focus of the current review is to highlight the advancements in the multicomponent systems processed using HME for the production of high-moisture meat alternatives (HMMAs). It specifically emphasises the impact of adding fibres to a protein-dense HMMA structure, the role of lipids and flavours, and their blending ratios (formulation). Additionally, the review delves into the crucial role of the extrusion parameters in the development of the product and the underlying mechanism of textural development that leads to phase separation. The study compiles the effect of various extrusion parameters on the quality of the HMMA, providing a detailed perspective yet simplified information on selecting the appropriate process parameters.

## 2. Microstructure and Macrostructure Formation during HME

The principles of structure formation mentioned below are interconnected and the previous reaction becomes a precursor for the succeeding. The structure formation can only be understood and controlled by taking the machine parameter settings, machine configuration, and ingredient properties into consideration. For further reading, the authors recommend the publication by van der Sman and van der Goot [32] on this topic.

### 2.1. Mixing of Dry and Wet Feed

Dry and wet ingredients must be mixed for even water partitioning, subsequently assisting in the uniform initial hydration of the feed material in the first section of an extruder. Different type of preconditioners might be used to assist in extending the hydration times. Biopolymers, specifically plant proteins and polysaccharides, experience the mechanical stress produced by the conveying and mixing elements of the screw, while the temperature is kept low enough (25–65 °C) to prevent protein denaturation or starch gelation with consequences for the melt viscosity. These thermomechanical stresses influence the protein dough structure and modulate the water distribution [33]. It is well known that wheat gluten (WG) bodies might be aggregated and cross-linked in a network by adding water, salt, cysteine, ascorbic acid, and a certain mechanical stress [34,35], while this observation does not apply to other plant proteins, wherein hydration and partial protein dissolution would be observed solely. The addition of oil and development of stable emulsion is assisted by using small droplets, high viscosity, and added or natural emulsifiers [36]. To reduce the oil coalescence and lubrication effects, the injection of pre-emulsions with the proteins or other surface-active ingredients has been suggested [37]. The “cold” mixture in the initial mixing zone of the extruder represents a multi-phase system (dispersion) with partially insoluble biopolymers and immiscible oil droplets.

### 2.2. Liquefaction and Aggregation

Following through the mixing stage, the biopolymers are subjected to increasing temperatures and shear from the screw movement in the subsequent extruder sections (barrels). This stage involves the hydration and continuous unfolding of the proteins, resulting in increased protein solubility and the reduced apparent viscosity of the melt, particularly in shear thinning substances [38]. The dissipation of heat through fluid mechanics is reduced, and additional heat must possibly be added through the external heating systems of the extruder barrel. It has been reported that protein aggregation and denaturation is a moisture-sensitive process which is highly affected by barrel temperature. The physical forces generated by shear are not known to contribute towards aggregation but instead assist in fragmenting these protein aggregates [39,40]. The moisture content affects material softening, cross-link density, and the formation of various intermolecular bonds, such as disulphide bonds, hydrogen bonds, salt bridges, and hydrophobic interactions [41]. The biopolymers thus create a transient network, with the formation of weak and strong and the breakdown of notably weak intramolecular and intermolecular bonds due to temperature and shear forces during HME. Even the partial breakdown of disulphide bonds has been speculated about, leading to the high reversibility of gel formation in HME [32,42]. High shear during protein aggregation might act selectively and result in atomic binding densification and the formation of more elastic gels.

### 2.3. Protein Alignment and Phase Separation

The alignment of protein aggregates using laminar flow is suggested to be crucial for protein structuring and mimicking muscle fibres [32,43]. Exceeding a critical shear rate in case of shear-thinning biopolymer melts is a condition for protein aggregate alignment [44]. Additional intermolecular bonds, mainly hydrophobic interactions and disulphide linkages, formed in the parts of the extruder and die with lower temperatures compared to the melting zone stabilize the structure of aligned proteins [32]. Strain-hardening of the aligned and cross-linked protein aggregates is expected to occur in the cooling die section.

### 2.4. Phase Elongation (Protein, Fat Droplets, and Gas)

Thermodynamic principles typically result in structures with minimal surface-to-volume ratios in the dispersed phase. The phase elongation during HME is obtained by using shear forces. A breaker plate (a hole-perforated plate) between the screw tips and the cooling die locally reduces the hydraulic diameter of the duct, potentially promoting phase elongation, influencing the memory effects of the melt [32,45]. The design of transition zones (conical and cylindrical, temperature instabilities, and the reduction or extension of the hydraulic diameter) could also influence the alignment of any dispersed phase, particularly if the mechanical properties of the continuous phase counteract the re-deformation of an elastic dispersed phase. The dispersed phase might undergo break-up and coalescence because of the extrusion shear forces. The literature suggests that the shear forces are comparatively small in the transition zone or extruder die and coalescence becomes a more likely phenomenon. An imbalance in the mechanical properties of phases composed of different biopolymers and caused by, e.g., differences in particle size and/or glass transition temperatures might cause imparity in their flow velocity [46], phase deformation, and phase separation [47].

### 2.5. Cross-Linking, Solidification, and Syneresis

Reducing the temperature and shear in a viscoelastic, pseudoplastic biopolymer melt favours cross-linking and solidification (gel hardening). During the solidification in the cooling die, the rate of intermolecular bond formation is considerably higher than bond breakdown, which is evident in the product characteristics, e.g., the elastic modulus [48]. The proteins’ botanical origin, the dry/wet protein extraction process, blends’ dry matter concentration, the temperature and shear profile in previous extruder sections, etc. influence the cross-linking density. Solidification can be controlled via heat removal from the extrudate’s outer surface while the process is limited by the low heat conductivity of the melt, resulting in characteristic cross-sectional temperature and viscosity profiles [38]. The cross-linking and solidification of proteins and other biopolymer melts can result in a reduced water holding capacity (Flory–Rehner theory), meaning water is released from the product, also known as syneresis [49]. Syneresis has been reported to promote lubrication, causing wall slipping effects within the extruder cooling die. The final extrudates’ deformation and thus structuring comes to a final point depending on the solidification (increasing inner forces in the extrudate) and the wall slipping effect (reduced outer forces between the extrudate and the die surface). It is proposed that the shape of the temperature profile in the cooling die influences the macroscopic structure of the meat alternative due to the considerable link between rheology and temperature.

## 3. Effect of Process Parameters on Protein Restructuring

Optimisation of the extrusion parameters is a crucial step to developing HMMAs. Various factors such as the type of extruder, processing temperature profile, screw configuration, type of die, and cooling die temperature influence the protein restructuring on different-length scales. Co-rotating intermeshing twin-screw extruders provide mixing, heat transfer, and pumping behaviours suited to handling short process windows efficiently such as dispersions with water, low- or high-viscosity protein solutions (melts), or even oil-in-water emulsions with an added lubrification effect. Combined with the adaptability of the screw configuration, twin-screw extruders have unmatched performance and capacity in manufacturing meat alternatives. In the following Section 3.1, Section 3.2 and Section 3.3, we discuss opportunities in the barrel temperature profile, screw configuration, screw rotational speed, and the cooling die settings and create a framework for understanding the related protein reactions (Table 1).

### 3.1. Temperature Profile of the Barrel

The extruder barrel consists of individually temperature controllable sections, with electric or compressed water heating and cold water cooling. The sections are screwed or clamped, providing thermal coupling and excessive heat conduction. Also, these barrel sections for twin-screw extruders are available in basic configurations which allow an inlet port for liquid feed and solid feed, venting, and closed segments [55]. Extruder barrels are technically identified based on their length-to-diameter (L/D) ratio. The L/D ratios used in the twin-screw extruder for meat alternative manufacturing include 24/1 [10,50], 29/1 [21,56], 40/1 [53,57], and 44/1 [58,59], though it is not limited to these combinations. An HME machine should be long enough to compress the protein dough to push it through the cooling die, and with a higher L/D ratio, the throughput can be increased while retaining similar cooking times. Therefore, relatively high L/D ratios are preferable in HME.

The design and temperatures are integral operational elements of the barrel. For obvious reasons (high pressures, safety, etc.), the sectional design of a barrel cannot be changed or modified; however, the temperatures can be altered in different zones of the barrel, and in increments also the length of the barrel, hence the L/D ratio for a certain machine. The barrel temperatures for HMMAs develop from 20 °C at least in the feeding zone [58] up to 170 °C in the cooking zone [11,50,56]. It is crucial to attain or exceed a critical temperature to activate the gelling potential of the ingredients, often denoted as “melting point”. This temperature cannot be detected directly in the extruder barrel. To predict temperature–time- and shear–time-dependent hydration, unfolding, dissolution, aggregation, alignment, and phase elongation, the rheological properties of individual plant proteins and their combinations must be determined, preferably in extrusion-like conditions. Several extrudate properties indicate the thermal history experienced by the protein. It has been reported that extrusion of pea protein isolate (PPI) at 170 °C decreases the protein’s solubility due to the formation of iso-peptide bonds. A more developed anisotropy was observed for a barrel temperature of 150 °C and a corresponding dough temperature of 120 °C [11]. Blends of PPI with oat protein required higher dough temperatures to obtain a suited anisotropy (130–140 °C) and the temperature increase correlated positively with the cutting strength of the extrudate [11]. The advanced denaturation of soy protein at a 170 °C barrel temperature influenced positively the water holding capacity of the extrudate [60]. Other techno-functionality indicators elucidating the thermal history of a protein are the water distribution, the protein network density, or the degree of syneresis.

HME favours the formation of covalent bonds (disulphide bonds) and non-covalent bonds (hydrophobic interactions and hydrogen bonds), among which disulphide bonds are thought to play a crucial role in the extrudate’s texture and structure [61]. Section 4 of the review will cover this aspect of protein chemistry.

### 3.2. Mechanical Effects through Screw Configuration and Rotational Speed

Extruder screws could be one-piece or formed with a union of small segments assembled over a shaft. One-piece screws are commonly used in single-screw extruders, whereas twin-screw extruders comprise an assembly of screw elements. The design or arrangement of the screw elements is largely responsible for their function, such as metering, intensive mixing/heat transfer, filling, and metering–compressing [55]. Altering the screw configuration (the forward and reverse elements) also affects the specific mechanical energy (SME) consumption and mechanical energy dissipation, comparatively higher for combinations of mixing and reverse elements than for forwarding and mixing elements only [56]. It is essential that the mixing zones are allocated specific temperatures for the directed transition of biopolymers and that the filling degree in such zones is sufficiently high to achieve a high shear rate and efficient heat transfer from the barrel to the dough. Zhang et al. [62] used 21 different positionings of screw elements for the HME of defatted soy meal. The inclusion of these advanced design elements was found to be responsible for the rise in the process’s SME. A screw profile with a reverse helix, stagger angles, a flow-restricting capability, thin kneading discs, and longer element lengths has resulted in a significant improvement in the screw-filling degree more than 3 times over. As a result, there has been a significant increase in the SME of up to 1.5 times over.

Wu et al. [63] found the anisotropy index of a soy protein and gluten composite can be modulated by using a varying moisture content, screw speed, barrel temperature, and gluten content, with a local optimum between the minimum and maximum tested. They obtained a texturized HMMA at an optimum screw speed of 16 Hz (960 rpm), and at higher and lower screw speeds, this optimisation strategy did not apply and may have been overlaid by the viscosity reduction in the shear-thinning dough.

Similarly, during the HME of plant proteins, torque gives an indication of ingredient flow, inconsistency, surging, or plugging. In addition, any variable that modifies the extruder melt viscosity has a corresponding effect on the motor torque.

### 3.3. Cooling Die Configuration

The cooling die has the function of reducing the dough temperature to prevent water evaporation and control the viscosity or viscosity differences for the elongation of structural elements and layer formation. For cooling the dough, rectangular-shaped slit dies, with or without rounded edges, multi-channel dies, annular gap dies, and annular gap dies with rotating parts are described in the literature and explained later in this same section.

Reported cooling die temperatures for meat alternatives include 80 °C [56], 70 °C [10,31,51], 60 °C [59], 56 °C [58], 30 °C [11], 20 °C [14,54], and −10 °C [57]. However, the material temperature is different from the temperature of the cooling agent and varies as a function of the dough temperature reaching the die (98–138 °C) over the length, and the sectional area, with a maximum in the centre of the fluid (>85 °C) [57], demonstrates the limitation of heat removal by the low thermal conductivity of the dough and heat conduction as the only principle of heat transfer in the die. Numerical analysis of the temperature profile with finite element methods is not fully validated and requires interpretation since the suggested conditions for conductive heat transfer in a material with constant thermal properties, a strict laminar flow, and zero wall slip have a theoretical character in solidifying liquids.

Increasing the temperature difference between the cooling agent and the material (dough) increases the steepness of the sectional temperature and the viscosity differences in the material, as well as the stress applied for a hypothetical zero wall slip in the laminar flow [57]. The protein layers can be deformed using a laminar flow [57], or the anisotropy is more pronounced with higher temperature differences or lower coolant temperature, respectively [54]. Vice versa, higher coolant temperatures might reduce the internal stress in the material, leading to the toughening of protein–carbohydrate structures, which are supposed to be less disaggregated mechanically [61,64]. Resulting from the increase in the viscosity or cohesive forces in the material, lowering the coolant temperature from 80 °C to 0 °C linearly increments the backpressure measured in the screw tip region [56]. Interestingly, dead-stop trials revealed that the restructuring of protein continues in the transition zone between the extruder screw tips and the cooling die [31,57], and can be interpreted as a general effect of cooling independent of the flow profile in the cooling die.

A well-designed LCD should essentially have functions such as (a) providing sufficient cooling, (b) appropriate conductance, (c) product uniformity with aligned fibrous structure development, (d) the capability to monitor and control the product conditions (temperature and pressure), (e) easy cleaning, and (f) simple design. This section presents a discussion on the currently available LCD designs for the HME of alternative proteins.

#### 3.3.1. Slit Die

Single slit dies have smooth inner surfaces with elongated rectangular or circular orifices. These are jacketed with a cooling unit essentially connected after a transition zone to form a thin sheet of meat alternative. These dies have limited orifice dead volume, which limits their throughput, thus becoming a bottleneck for the commercial extrusion process. Moreover, the die design poses challenges in product flow as the outer layer of the extrudate tends to stick to the inner wall of the cooling die, leading to lowering the product conductance and negatively affecting the overall efficiency [65]. Multi-channel cooling dies were designed to eliminate the constraining factors of pre-existing cooling dies, such as limited throughput, the development of velocity, and shear gradient causing an inconsistent extrudate flow [65]. The literature search revealed that multi-channel dies comprising a set of parallel channels (i.e., 24 channels) with the dimensions 6–8 mm × 70–90 mm, a total length of 0.7–1.2 m, and throughput capacity of 1000 kg/h have been used at commercial production capacity.

#### 3.3.2. Annular Gap Die

Klein et al. [66] constructed an annular gap die, designed in a ring configuration offering uniformity in product distribution, flow velocity, as well as cooling (mainly via conductive heat transfer). The main elements of the die include the inlet and outlet ends, a thermally controlled jacket, a conical distributor, and an extrudate flow channel. Introducing an aperture into the region of the distributor (between the extruder and extrudate flow channel) could improve the fibrous structure range. Especially designed apertures such as pinholes could develop a product with a resemblance to beef, whereas slotted apertures could possibly develop a chicken- or fish-like texture [66]. It could function in a capacity of 125 to 2000 kg/h with a length of 0.5 to 2.5 m and a diameter of 100 to 800 mm.

#### 3.3.3. Rotating Annular Gap Die

Beyrer et al. [67] developed an annular gap die which was constructed to achieve well-defined shear and heat transfer, with a static outer cylinder and two rotating inner parts. The section next to the extruder aperture is generally kept at a higher temperature and shear in order to aid texture formation, whereas the following section of the die is operated at a lower temperature and shear to set the preformed fibrous structure. It was observed that the reorientation of protein layers or fibres might be achieved by applying additional shear to the dough in a rotational cooling die, where the main shear rate is independent on the overall material flow rate and hydraulic diameter of the die [42].

In general, the design of the cooling die elements allows for the imitation of different meat structures. A minimal cooling die aperture height is required to create the essential temperature gradient and velocity profile, thus developing a meat-like texture [68]. However, the increase in the aperture height should be in proportion to the extension of the cooling die length to ensure the development of a sufficient process SME, pressure, and torque to texturize the proteins. The cooling die design also has a substantial effect on the product conductance. Forte [69] simplified the conductance equations of conventional dies (assuming Newtonian flow). Increasing the aperture area enhances conductance while increasing the length of the cooling die negatively influences product conductance. The conductance of annular cooling dies is higher compared to slit dies. To bring it all together, annular dies with an appropriate balance of aperture height and die length possibly result in the development of highly texturized meat alternatives.

## 4. Effect of Different Ingredients on Fibrous Structure Formation

### 4.1. Proteinaceous Ingredients

Alternative proteins have had a profound large market share in recent years. The commercial and economical sources for the isolation of these proteins include plants, aquatic biomass, and insects. To have a higher recovery and attain high-quality proteins, novel green extraction technologies have been already explored [70]. In a study, Kumar et al. [71] detailed a review of physical pre-treatment (ultrasound, pulsed electric field, high pressure treatment, and microwave) and biochemical extraction methods (using single enzymes and concoctions of enzymes) for the isolation of plant proteins. Wet extraction followed by isoelectric precipitation and salt extraction produces protein isolates with a protein content of 81.9–88.79% and 74.71–81.98%, respectively. Commercially, these precipitates of proteins are spray-dried in order to obtain a fine powder. On the other hand, dry extraction for, e.g., air classification requires the fine milling of the cotyledon portion of pulse grains to completely disrupt the cells and fragments without damaging the starch granules severely. Further, fractionation via air classification produces protein concentrates (50–70%) as a fine fraction (rich in protein) and coarse fraction (rich in starch).

The reactivity and functionality of proteins are directly influenced by the botanical source and processing background (isolation/fractionation and purification). It is known that the protein isolates carry a higher portion of denatured proteins than native proteins, due to the pre-processing methods. It has been stated that denatured proteins require higher energy for their activation than their native counterparts. Thus, the conditioning (temperature and shear) requirements for the extrusion of soy, pea, faba, and other protein isolates are inherently different from protein concentrates and vital gluten.

#### 4.1.1. Soy

Soy (*Glycine max*) protein isolates and concentrates have been used as an alternative protein source for decades, owing to their capacity to create a cross-linked fibrous network, making them an apt ingredient for meat alternative development. Soy protein could be classified into four types: 2S, 7S, 11S, and 15S. Their specific composition is controlled by factors such as soybean variety, growth conditions, and pre-processing procedures [21,72]. 7S and 11S accounts for almost two-thirds of total soy protein, with globulin accounting for most of 11S as well as one-half of the 7S fraction [72,73]. The globulin present in both protein fractions form disulphide-linked polymer, causing an increase in viscosity and a reduction in protein solubility. Heating of soy protein under moist condition leads to the formation of cross-linked gels, which are attributes of disulphide bonds and sulfhydryl–disulphide interchange, contributing to the stabilisation of gel network [72].

SPI with moisture levels ranging from 20 to 70% (dry basis) melts at 140 °C and its flow behaviour is strongly dependent on the moisture content, causing free water to act as a lubricant in high-moisture conditions, i.e., 54–70% (dry basis) [74]. However, high shear and the resulting pressure development during extrusion favour the melting of soy proteins at relatively lower temperatures.

Extrudate’s developed with only SPI and no other biopolymer exhibited a phase separation phenomenon, revealing one protein-rich and another water-rich dispersed phase [21]. The extrusion temperature showed no effect on an extrudate’s solubility in deionised water, and it was substantially greater than the raw material, implying that extrusion does not influence disulphide bond formation, which tends to reduce the protein solubility.

According to Fang et al. [75], raising the SME (achieved by changing the screw profile) improved SPI’s solubility in phosphate buffer while also increasing the proportion of low-molecular-weight protein components. The SME of an extrusion process represents the total mechanical energy required and is largely influenced by the feed material properties, feed rate, screw profile and speed, and extrusion temperature conditions. A higher SME in SPI extrusion indicated rigorous processing conditions which affected the textural properties (increased tensile strength and hardness), decreased the dough viscosity in the die, and produced an extrudate with a darker colour.

Development of fibrous networks in plant proteins essentially requires a sufficient moisture supply, mixing, a high shear and temperature, and an additional attachment, i.e., a cooling die, to contain these fibre network in the end product. Chen et al. [76] developed SPI meat alternatives in a wide spanning range of low to high moisture and different cooking temperatures. Altering the cooking temperature was shown to have a nominal effect on the product characteristics compared to changing moisture content. A higher extrusion moisture content lowered the dough viscosity, residence time, and energy conversion (mechanical to heat), resulting in a significantly reduced tensile strength, hardness, chewiness, and degree of aggregation.

A major challenge to soy proteins’ global acceptability is difficulties in their cultivation in the colder climate of northern Europe and people being allergic to these proteins. To minimise our reliance on it and foster understanding about other potential ingredients, it was essential to substitute soy protein (partially or completely) with other protein sources. Zahari et al. [52] incorporated hemp protein concentrate in different ratios to determine the critical blending ratio for achieving a fibrous texture. While blending such different proteins, it is crucial to consider their functional, rheological, and thermal properties since they play a major role during mixing, liquefaction, phase separation, as well as cross-linking and solidifying. The results of this study showed that hemp protein concentrate required a longer time and higher temperature to bind water and denature proteins than SPI [52]. Furthermore, as the make-up of the proteins is different, the melting temperature for both the proteins were in different ranges, 90.3–123.1 °C for the hemp protein concentrate and 54.6–107.0 °C for the SPI. Similarly, Schreuders et al. [77] prepared mixtures of SPI/PPI with vital WG and obtained fibrous structure at varying extrusion conditions. Wittek et al. [53] developed meat alternatives by blending SPI and whey protein concentrate (WPC) in different ratios. The anisotropic index was significantly improved for the meat alternative developed with a higher WPC content; the structures were more disordered, shorter, and thinner, and the V-shape angle decreased with an increasing WPC concentration. Furthermore, there are several other ingredients blended with SPI to develop meat-like fibrous structures. However, it must be emphasised that differences in ingredient properties could largely influence amalgamation and lead to erratic material flow and product development.

#### 4.1.2. Gluten

Vital WG is produced by isolating protein (basically gluten) from wheat in its native or reversibly denatured state [78]. The hydration and mechanical mixing of vital gluten causes gluten network formation by modifying the initial arrangement of gliadins and glutenins. On hydration, it can quickly regain its viscoelastic properties (elasticity and extensibility) through the unfolding of protein molecules favouring the water distribution (heterogeneous) around the long protein chains, particularly near charged ionisable groups and polar amino acids [79].

The polymerisation of vital gluten begins with hydration, mixing, and slight heating in the initial extruder barrel. It modifies the rheological properties of the protein melt further in the melting zone, resulting in the development of anisotropic product textures [80]. Thus, control of the polymerization reactions taking place in the screw section is necessary to control the characteristics of meat alternative products. Since vital gluten can develop a rubbery and elastic network at relatively lower activation energy, extensive shearing in the later zone could result in protein depolymerisation (the breakdown of polymer chains due to mechanical stress) [81]. Thus, introducing additional mixing elements in the later zone could lead to disintegrating the continuous gluten fibre network.

Pietsch et al. [56,80] used a single mixing (or kneading) element for the structuring of vital gluten. They observed the considerable impact of changing the feed rate and screw speed on protein extractability (under reducing and non-reducing conditions). Protein extractability is largely governed by the polymerisation and depolymerisation of gluten during extrusion. Additionally, increasing the barrel temperature clearly decreased the protein’s extractability, indicating the formation of covalent bonds (such as disulphide bonds). Moreover, development of anisotropic structures, as well as an increase in hardness and Young’s modulus, could be connected with an increase in WG polymerization [82].

#### 4.1.3. Pea

Pea (*Pisum sativum*) is one of the world’s most produced legume crops, contributing 26% of the total world legume production [83]. Although it is a relatively new ingredient, it has profound food applications in products such as meat and dairy alternatives, baking, dressings, beverages, emulsions, etc. Pea has a protein concentration of 20–25%, classified into four major groups: globulin, albumin, prolamin, and glutelin [83,84]. Among them, globulin and albumin are the major storage proteins, globulin alone contributing to 65–80% of PPI. It is composed of legumin 11S and vicilin 7S, with their ratios ranging from 0.2 to 1.5 depending on genetics and processing [85]. Gelation, being an essential function of globular proteins, plays an important role in products’ texture development. The thermal gelation of pea protein is influenced by various factors, such as the cultivar, protein heterogeneity, isolation methods, solvent, and heating parameters [86,87]. HME conditions favour the gelation and functional modification of pea proteins in developing anisotropic structures.

Thermal scanning of native PPI reveals two major endothermic peaks at 67.1 and 85.1 °C. The first peak might be due to the thermal transition of non-globular fraction (starch and/or fibre), whereas the second peak could be due to the denaturation of the vicilin and legumin fractions [88]. Thermographs of commercial PPI show a single endothermic peak at 68.2 °C, suggesting denatured protein fractions as a result of the isolation process. Thus, it is important to consider the protein isolation process as one of the factors to alter the protein functionality.

Zhang et al. [89] developed PPI-based meat alternatives at varying moisture contents to understand its impact on functional and structural properties. The secondary and tertiary structures of PPI extrudates were considerably changed, altering their functional properties. Protein aggregation during extrusion resulted in a decreased solubility and foaming capacity.

Interestingly, different commercial PPIs with a similar basic chemical composition show differences in their thermal and rheological properties [38]. Authors have suggested that the PPIs with bigger particle sizes were easier to process in an extruder than isolates with smaller particle sizes.

Wang et al. [15] reported that hydrophobic interactions and hydrogen bonds were more important in stabilising the fibrous structure than disulphide bonds (which are generally considered important in the development of fibrous structures during HME). They obtained pea protein extrudates with greater resemblance to chicken breast than the extrudates produced using SPI under the same processing conditions, since SPI formed compact extrudates with a hard and rubber-like texture.

#### 4.1.4. Faba

Faba beans (*Vicia faba* L.) are legume crops rich in protein (32–36%), carbohydrate (44–47%), dietary fibre (~8%), and ash (3.5–4%), as well as a good source of micronutrients such as antioxidants, γ-aminobutyric acid, and phenols [90,91]. They have globulin as storage proteins, including the 7S and 11S fractions, with molecular weights ranging from 48 to 75 kDa and 20 to 30 kDa, respectively, but lack sulphur-containing amino acids (cysteine and methionine) [90,92]. Faba bean protein concentrate (86.6%, protein content) has a thermal denaturation temperature of 88 °C, slightly higher than the field pea and lower than that of soy protein. In comparison to soy, faba beans have the potential to grow in cold climatic conditions, are effective in biological nitrogen fixation, and are an underutilized crop to meet the growing need for soy alternatives.

Combining faba protein isolates and concentrates with varying die temperatures and feed moisture contents greatly influences fibrous structure formation [93]. Since protein favours the production of cross-linked structures, recipes with a higher content of faba protein isolates create less orientated fibrous structures, and brittle products due to the formation of a tighter protein network. On the other hand, faba protein concentrates having a considerably higher content of polysaccharides favours phase separation, leading to them forming more pronounced and oriented fibrous networks.

Ferawati et al. [54] produced HMMAs using wet extracted (alkali soluble) protein isolates from yellow peas and faba beans to evaluate their potential as soy protein replacement ingredients. The faba beans produced an anisotropic structure at a relatively lower temperature than yellow pea protein, which corresponds to their lower denaturation temperature, leading to early melt, realigning the protein molecules, and favouring the structure formation.

It has been reported that faba protein concentrate (63.5% protein content) can be solely used for the structuring of meat alternatives [14]. The anisotropic index of these developed meat alternatives decreased with an increasing temperature, feed rate, and moisture, which might be due to overcooking, a shorter retention time, and an excessive softening and lubrication effect.

The available scientific literature strongly suggests that faba protein concentrates could be considered as better ingredient for HMMA development due to the possible phase separation of protein and polysaccharides. Reducing the degree of protein refining substantially reduces the carbon footprint [94], thus encouraging the use of faba protein concentrates over isolates.

### 4.2. Other Ingredients and Their Combinations

In order to minimise the environmental impact occurring due to the consumption of animal meat, the novel approach of using plant-, insect-, and animal-based ingredients together is also gaining popularity. Using compound protein ingredients could be effective in reducing the dependence on animal meat for good-quality protein, which might reduce global animal meat consumption and subsequently result in a reduced environmental impact. The idea of using a combination of ingredients from diverse sources is enhanced nutritional and functional attributes, improved organoleptic properties, and minimised production costs of products. Caporgno et al. [58] studied the effect of incorporating microalgae (*Auxenochlorella protothecoides*) biomass into soy protein concentrate. The combination of ingredients changed the fibrous structure formation, possibly due to microalgae’s higher fat content and highly resistant cell wall. However, the developed product was nutritionally rich and attained an attractive colour. The incorporation of microalgae as an ingredient in meat alternatives has been reviewed in detail elsewhere [95]. Blending egg white protein powder (EWPP) with a WG/S combination to enhance the anisotropic structure formation resulted in increasing the WG aggregation at a concentration of 2% EWPP and 98% WG/S [50]. Furthermore, EWPP incorporation enhanced the disulphide bonds and cross-linking.

Blending different ratios of SPI with whey protein concentrates to produce a HMMA resulted in a product with a pronounced anisotropic index [53]. Among all the products, the ratio of SPI to WPC (70:30) resulted in the highest anisotropic index of 1.60. It was observed that under a constant mass flow rate and die geometry for SPI:WPC blends, the die pressures were directly proportional to the material melt viscosity. This may have been due to a) a simple combination of rheological properties derived from the mixing of individual ingredients or b) a dispersed phase effect due to the mixing of incompatible ingredients (which results in the weakening of the overall structure) [96].

Over the past few years, there has been a growing trend of using insect proteins due to their numerous benefits, such as high levels of protein, vitamins, and minerals, as well as being an environmentally sustainable and cost-effective protein. Considering all the benefits of addressing food security issues as the world population continues to grow, Smetana et al. [97] developed meat alternatives using a combination of soy and insect (*Alphitobius diaperinus*) protein concentrates. The extrudate retained a fine, porous, and directional texture on incorporation of up to 30% insect protein concentration, which on further increase led to a more bulky texture. However, the product’s success is challenged by consumer acceptance, expensive processing, and environmental impact (higher than plant-based proteins, but much lower when compared to animal-based proteins). Popularly known edible insects explored for protein extraction include larvae, beetles, grasshoppers, crickets, termites, bees, and dragonflies [98].

### 4.3. Fibre-Rich Ingredients

As outlined in the above sections, having different biopolymer phases certainly favours the anisotropic structure development in HMMAs. Thus, it is advantageous to have protein and polysaccharide in appropriate ratios which are sufficiently processed during HME to obtain the desired product characteristics. The common structuring ingredients include starches, alginates, fibre, pectin, carrageenan, methylcellulose, and gums [99]. The addition of polysaccharide modifies blends’ rheological properties, such as soluble starches (potato starch), which tend to cause a decrease in viscosity, thus leading to lesser fibrous structure formation [100]. On the other hand, enriching the blend with non-starch polysaccharide (κ-carrageenan and curdlan) promotes molecular aggregation, thus resulting in the improved hardness of the product. This suggests that the differences in the conformational structure and composition of polysaccharides (starch/non-starch) has a significant impact on the textural characteristics of HMMAs. Also, blending two or more structuring ingredients together could be used to attain a synergistic effect, but this will further complicate the understanding of ingredient functions.

The formulation plays a crucial role in fibrous structure formation. Starches are exogenous polysaccharides which could promote meat-like structure development when added within limits [51]. Zhang et al. [101] have established the relationship between starch gelatinisation and the textural properties of soy protein and different starch extrudate systems. According to the above study’s findings, higher blend enthalpy changes result in higher pressure and SME fluctuations during extrusion, which causes a rise in product hardness and tensile strength, and successively leads to fibrous structure degradation. The fibrous indexes (ratios of lengthwise to crosswise strength) of SPI and WG with different starch blend extrudates were evaluated and found to be in the following order: corn amylopectin (1.40) > wheat starch (1.31) > cassava starch (1.24) > potato amylose (1.23) > potato starch = sweet potato starch (1.18) > corn starch (1.15) > mung bean starch (1.12) > pea starch (1.09) [101].

Amylose and amylopectin are conventional structuring ingredients used in food processing. Structuring a meat alternative by mixing PPI with amylose or amylopectin creates layered gel-like or cross-linked structures, respectively. Amylopectin incorporation increases the α-helix with the loss of β-turns, whereas amylose causes phase separation at a later stage than amylopectin, which intensifies protein self-association and aggregation in the die, hindering fibrous structure formation [10].

Zhang et al. [51] determined the effect of blending peanut protein powder with different exogenous polysaccharides on anisotropic structure development. Among carrageenan, sodium alginate, and wheat starch, sodium alginate (0.1%)-based extrudates were found to retain the highest fibrous index (1.24), but also increased the hardness and chewiness of the product. Moreover, it was observed that the carrageenan-based extrudates were not conducive to fibre orientation and increasing the amount of wheat starch (0–8%) in extrudates lowered the fibrous index [51]. Blending sodium alginate (0.1%) and wheat starch (2%) caused secondary structural changes in the peanut protein powder in the following order (β-sheet > β-turn > α-helix > random coil).

Next-generation meat alternatives are designed to increase fibre portions in the human diet. Incorporating oat fibre concentrates into PPI reduced the mechanical strength and void fractions and also negatively affected the fibrous structure [64]. Thus, it becomes essential to enhance the fibre content in a way that also preserves the fibrous structure of the product.

*Haematococcus pluvialis* is a commercial source of natural astaxanthin extraction; however, residues are not valorised or generally used for animal feed. Incorporating these residues enhanced the extrudate colour (comparable to red meat) and also improved the fibrous index (to 1.28 with maximum at 10% incorporation) [59].

In a study, Sakai et al. [102] determined the synergistic effect of incorporating sugar beet pectin and laccase into texturized pea protein granules to develop meat alternative patties. The combination of these additives resulted in a product with better colour and functional properties. Table 2 summarises the impact of different formulations on the meat alternative structure development.

### 4.4. Lipid, Colour, Flavour and Other Additives

Plant protein ingredients such as SPI have off flavours, i.e., beany and grassy flavours developed due to the peroxidation of unsaturated fatty acids, and astringent and bitter flavours due to the presence of isoflavones and saponins [105,106]. Alternative protein-based meat alternatives also have a lower fat and heme (precursor of haemoglobin) content, which makes it difficult to meet an animal-meat-like tenderness and flavour [20,107]. Though it is interesting that the complex chemical sites of plant protein are considered to be sound for the adsorption of flavouring compounds, the extensive use of synthetic flavouring compounds is not promoted. Meat alternatives, in most cases, are highly processed foods due to the inclusion of various purified ingredients obtained from diverse sources, which are further processed to mimic meat’s taste and structure. The consumption of synthetic colours, flavours, and other additives has a teratogenic effect on consumption over a long period; thus, this must be replaced within our daily diet for better human health [108]. Hence, it is important to consider the health implications of the lipids, flavours, and colours being added to attain meat-like attributes.

Lipids improve the flavour as well as maintaining the texture, juiciness, and mouthfeel of food products. Animal-based meat products are rich in lipids with a typical distribution in different parts of their muscles [109]. Traditional HME-based meat alternatives have been constructed with a relatively lower lipid content. However, modern meat alternatives attempt not only to balance protein but also enrich them with lipids in almost equal ratios as found in animal meat. Popular plant-based lipids for meat alternative development are sourced from seeds and nuts (27%), soybean oil (18%), olive oil (10%), sunflower oil (8%), hydrogenated fat (6%), palm oil (6%), and coconut oil (2%) [110]. Cocoa butter, canola oil, and corn oil are the other commercial lipids being used [111]. The use of semi-solid fats such as coconut oil and cocoa butter, which are also rich in saturated fat, is criticized due to the associated health risk [112]. Incorporation of lipids at a higher concentration could cause a lubrication effect, which will limit the feed-to-barrel pressure during HME and hence lead to developing a less integrated product. To this, emulsions could be a solution: Wang et al. [37] improved the oil content up to 8% from 4% in SPI-WG extrudates through the use of emulsions. Thus, the selection of lipid, preparation method, and concentration of the emulsion plays a crucial role.

Meat’s flavour plays a crucial role in selection, quality evaluation, and consumer acceptance, which could be evaluated using chemical analysis methods and human sensory perception. On human consumption, olfactory senses detect the meat aroma prior to consumption, flavour aromatics are sensed while chewing, mouthfeel is sensed by trigeminal receptors, and aftertaste is perceived after swallowing. The mechanism of the flavouring compounds being released is complex, and thus requires a better understanding of their intrinsic nature, concentration, and interactions with other components of food [113]. The interaction between proteins and flavour compounds is influenced by the aliphatic chain length (an increase in the aliphatic chain in homologous compounds leads to higher flavour retention). Flavour compound adsorption in proteins is also governed by reversible non-covalent forces such as electrostatic and hydrophobic interactions, and hydrogen bonding (which usually forms during HME) [114]. Moreover, flavouring compounds also enhance the protein binding capacity, subsequently leading to enhanced fibrous structure formation. Zhang et al. [107] studied the adsorption capacity of different flavouring compounds (carbonyl → 2-octanone, alcohol hydroxyl → 1-octen-3-ol, and aldehyde group → octanal) in the SPI solution. Their results suggest that the octanal has the highest binding affinity towards the SPI, which might be due to the stronger hydrogen forces between the SPI and the aldehyde group.

Colour is an important attribute of food which is often considered by most consumers when they purchase a product. Primarily, myoglobin is responsible for the characteristic colour of meat products, which undergo chemical changes during their processing and cooking [115]. Similarly, different ingredients and their HME during meat alternative development impart characteristic colours on the end product. Plant-based colours such as beetroot juice extract (Beyond burger), soy leghemoglobin (Impossible meat), and lycopene from tomato (MorningStar Farms burger) are commercially being used in the development of meat alternatives [111].

## 5. Conclusions

An increasing demand for alternative protein ingredients among consumers has challenged food processors to replace animal proteins in product development. Extrusion being one of the common methods in food manufacturing at an industrial scale, it is an easy-to-adopt technique to produce animal-meat-like textured products. Although structure formation during HME is achieved by using a combination of different mechanisms, they can be guided by changing the extrusion processing parameters. A high barrel temperature increases the protein polymerization reactions by enhancing the degree of protein denaturation, whereas shear forces cause the continuous aggregation and fragmentation of these denatured proteins. A lower cooling die temperature favours the formation of a layered structure, and leads to molecular restructuring within the denatured protein molecules. Amalgamating alternative protein sources in combination with other biopolymers, flavours, and colouring agents is essential to obtain the desired product qualities. However, in-depth studies are recommended to determine the intestinal digestibility and absorption of such protein–biopolymer blends. Meat alternatives are in-demand products which require constant research through exploring new ingredients and the integration of technologies.

## Figures and Tables

**Table 1:** Effect of HME process parameters on the development of plant-based meat alternatives’ structure.

Ingredients	Extrusion Process Parameters	Texturization/Anisotropic Index	Mixing/Kneading Elements	Effect of Extrusion Parameters	Reference
Peanut protein biomass waste	Feed rate (6 kg/h), feed moisture content (55%); Screw speed (210 rpm); Extruder barrel (red) and cooling die (blue) temperatures: 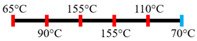	1.10	8	Fibrous structure formation started at the die and cooling zone junction. The α-helix/β-sheet ratio was highest in the final extrudate than during other zones of extrusion.	[31]
EWPP and WG/S	Feed rate (10 kg/h); Screw speed (270 rpm), diameter (36 mm) and L/D (24:1); Extruder barrel (red) temperatures: 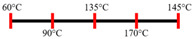	2.50	-	Heating during extrusion in the presence of EWPP supported the polymerization of WG.	[50]
PPI and amylose/amylopectin	Feed rate (6 kg/h); Screw speed (240 rpm) L/D (24:1); Extruder barrel (red) and cooling die (blue) temperatures: 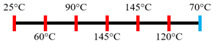 Cooling die dimensions (70 × 10 × 1800 mm^3^).	-	-	Protein–protein and protein–amylopectin interactions were favoured in the cooling zone (70 °C).	[10]
SPI and PPI	Feed rate (0.3 kg/h); screw speed (200 rpm); Extruder barrel (red) and cooling die (blue) temperatures: 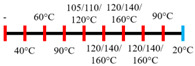 Cooling die dimension (5 × 20 × 200 mm^3^).	-	-	Changing barrel temperature least affected the textural characteristics of PPI. Increasing the barrel temperature enhanced the anisotropic structure formation.	[15]
Pea protein powder, carrageenan, sodium alginates, and wheat starch	Feed rate (6 kg/h); feed moisture (55%); Screw speed (210 rpm); Extruder barrel (red) and cooling die (blue) temperatures: 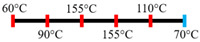	1.24	-	The cooling zone favoured the formation of hydrogen and disulphide bonds and their interactions to maintain the protein structure.	[51]
Hemp protein concentrate and SPI	Feed rate-solid (0.8–1.1 kg/h) and liquid (1.88–2.36 kg/h); Screw speed (300, 400, 500, 600, 800 rpm), diameter (20 mm); Extruder barrel (red) temperatures: 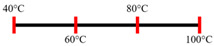 Cooling die dimension (25 × 7 × 300 mm^3^).	-	5	Hemp protein concentrate as a substitute protein source with SPI yielded a more acceptable structured product at a screw speed of 800 rpm. However, SPI alone extrudates were developed at 500 rpm speed. The barrel temperatures 40, 60, 80, and 100 °C were selected over 60, 80, 100, and 120 °C in the preliminary trails.	[52]
PPI and oat protein concentrate	Feed rate solid (300 g/h) and water (330 mL/h); Screw speed (300 rpm), diameter (11 cm); Extruder barrel (red) and cooling die (blue) temperatures: 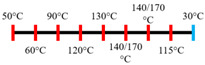 Cooling die dimension (4 × 20 × 250 mm^3^).	-		The PPI extrudate obtained a fibrous structure with a smooth surface at an extrusion temperature > 150 °C and melting temperature > 120 °C. The PPI and oat protein concentrate mixture required a higher extrusion temperature (170 °C) and melting temperature (140 °C). Protein extractability was reduced.	[11]
SPI	Feed rate-solids (5 kg/h) and water (5 kg/h); Screw speed (250 rpm), L/D (29:1); Extruder barrel (red) and cooling die (blue) temperatures: For material temperature 124 °C 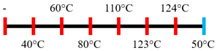 For material temperature 135 °C 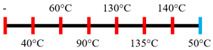 Cooling die dimension (380 × 30 × 6 mm^3^).	-	3	With an increasing material temperature (124 to 135 °C), the anisotropic structure of the extrudate was improved. Solubility in deionized water increased at both extrusion temperatures → indicating the disruption of non-covalent interactions.	[21]
SPI and whey protein concentrates	Feed rate protein blend (0.9 kg/h) and water (1.1 kg/h); Screw speed (600 rpm), diameter (11 mm), and L/D (40:1); Extruder barrel (red) and cooling die (blue) temperatures: 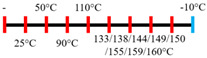 (Adjusted to attain a final material temperature of 115 °C, 125 °C, and 133 °C) Cooling die dimension (125 × 19 × 4 mm^3^).	1.60	0	Increasing whey protein concentration negatively affects die pressure and reduces the multi-phase morphology ‘V-shape’ angle. Increasing the final material temperature from 115 °C to 133 °C enhanced the anisotropic structure formation.	[53]
Yellow PPI (both commercial and local *) and faba bean protein isolate (both commercial and local *)	Mass flow rate (2 kg/h); Screw speed (400, 600, and 800 rpm), diameter (20 mm), and L/D (40:1); Extruder barrel (red) and cooling die (blue) temperatures: Yellow PPIs 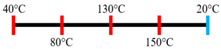 Faba bean protein isolate 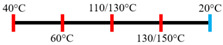 Cooling die dimension (7 × 25 × 300 mm^3^).	-	5	Increasing the temperature to 130 °C and 140 °C of the zone (3 and 4, respectively) enhanced the fibrous structure’s formation. The extrusion temperature must be sufficient for ingredients to melt to form the fibrous structure. The SME of extrusion decreases with an increasing barrel temperature. A prominent fibrous structure with yellow PPI (local) was obtained at 67% moisture content and a screw speed of 400 and 600 rpm. The faba bean protein isolate (local) formed a fibrous structure at 62% moisture content, screw speed (800 rpm), and temperature (40–60–110–130 °C), resembling boiled chicken and beef.	[54]
Faba bean protein concentrate	Feed rate-product (0.95 to 1.30 kg/h) and the ratio of water-to-product feed rate (3.0 to 5.0); Screw speed (900 rpm); Extruder barrel (red) and cooling die (blue) temperatures: 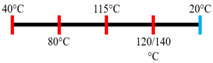 Cooling die dimension (30 × 5 × 300 mm^3^).		4 × Forward mixing element; 1 × Reverse mixing element	An increasing moisture content and barrel temperature reduced the SME. At a higher barrel temperature, an increasing feed rate increases the die pressure. Higher water content, feed rate, and extrusion temperature decreased the anisotropic index of the extrudate. The anisotropic index was at a maximum for the intermediate SME. Higher SME application causes strong network formation but lacks in the anisotropic index. Best processing parameters: the ratio of water-to-product feed rate (4.0), product feed rate (1.10 kg/h), and zone-4 temperature (130 and 140 °C).	[14]

* Local refers to protein isolate obtained by the author in the lab using the chemical method. L/D, length-to-diameter ratio; EWPP, egg white protein powder; PPI, pea protein isolate; SPI, soy protein isolate; SME, specific mechanical energy; WG/S, wheat-gluten-to-starch ratio.

**Table 2:** Effect of ingredient combinations on the development of HME-based plant-based meat alternatives’ structure.

Protein Ingredient	Structuring Ingredients, Flavouring and Other Ingredients	Extrusion Moisture Content/Feed Rate	Product Description and Image (of Best Resultant Combination)	Reference
WG (9 portions), EWPP (0.25, 0.5, 0.75, 1, and 2%)	Wheat starch (1 portion)	30%	EWPP (2%) 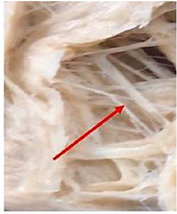	[50]
WG (9 portions)	Starch (1 portion), phosphates (sodium tripolyphosphate, sodium pyrophosphate, sodium hexametaphosphate) (0, 0.25, 0.5, 1, and 2%)	30%	Sodium tripolyphosphate (2%) 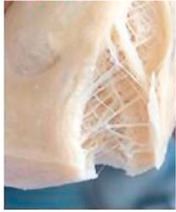	[13]
PPI (9 portions)	Amylose (1 portion)/amylopectin (1 portion)	58%	Amylopectin (10%) 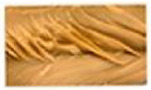	[10]
SPI/PPI (19.5%) and WG (19.5%)	Sodium chloride (1%)	60%	SPI (19.5%) and WG (19.5%) at 120 °C 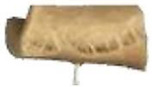	[77]
SPI (100, 95, 90, 80, 70, 60, and 50%)	Microalgae powder (0, 5, 10, 20, 30, 40, and 50%)	55, 60, and 65%	SPI (70%), microalgae powder (30%) and moisture content (60%) 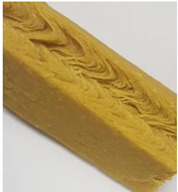	[58]
Soy protein concentrate (planetary roller extruder)	Iota (ι) carrageenan (ICGN) (0, 0.75, 1.5, 2.25, and 3%)	10 kg/h	ICGN (2.25%) 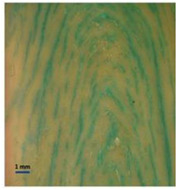	[103]
Isolated soy protein (50%), WG (40%)	Corn starch (10%)	700 g/kg	Isolated soy protein (50%), WG (40%), corn starch (10%), screw speed (200 rpm) 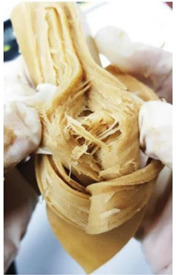	[104]

EWPP, egg white protein powder; PPI, pea protein isolate; SPI, soy protein isolate; WG, wheat gluten.

## Data Availability

The data used to support the findings of this study can be made available by the corresponding author upon request.
